# Supplementary material for: Intracellular Penetration and Effects of Antibiotics on Staphylococcus aureus Inside Human Neutrophils: A Comprehensive Review
Source: Antibiotics (Basel). 2019 May 4;8(2):54. doi: 10.3390/antibiotics8020054 (PMC6628357; doi:10.3390/antibiotics8020054)
Supplement: Supplementary file 1 [file antibiotics-08-00054-s001.pdf]

1. Search String

1.1. PubMed Search String

("Anti-Bacterial Agents"[Mesh] OR anti bacterial agent*[Title/Abstract] OR antibacterial agent*[Title/Abstract] OR antibiotic*[Title/Abstract] OR bactericidal agent*[Title/Abstract] OR anti-biotic*[Title/Abstract] OR Anti-microbial drug*[Title/Abstract] OR antimicrobial drug*[Title/Abstract] OR anti-microbial agent*[Title/Abstract] OR antimicrobial agent*[Title/Abstract] OR anti-microbial therapy[Title/Abstract] OR antimicrobial therapy[Title/Abstract] OR "Ceftriaxone"[Mesh] OR Ceftriaxon*[Title/Abstract] OR Rocephin*[Title/Abstract] OR "Amoxicillin-Potassium Clavulanate Combination"[Mesh] OR amoxicillin clavulanic acid[Title/Abstract] OR amoxicillin/clavulanic acid[Title/Abstract] OR Amoxicillin Clavulanate[Title/Abstract] OR Augmentin[Title/Abstract] OR "Ciprofloxacin"[Mesh] OR Cipro*[Title/Abstract] OR "Clindamycin"[Mesh] OR Clindamycin*[Title/Abstract] OR Dalacin C[Title/Abstract] OR "Gentamicins"[Mesh] OR Gentamicin*[Title/Abstract] OR "Vancomycin"[Mesh] OR Vancomycin*[Title/Abstract] OR Vancocin*[Title/Abstract] OR "Penicillin G"[Mesh] OR "Penicillin G"[Title/Abstract] OR Benzylpenicillin*[Title/Abstract] OR "Floxacillin"[Mesh] OR Floxacillin*[Title/Abstract] OR Flucloxacillin*[Title/Abstract] OR Floxapen[Title/Abstract] OR "Rifampin"[Mesh] OR Rifampi*[Title/Abstract] OR Rifadin*[Title/Abstract] OR "Cefazolin"[Mesh] OR Cefazolin*[Title/Abstract] OR Cephazolin*[Title/Abstract] OR Kefzol[Title/Abstract]) AND ("Intracellular Space"[Mesh] OR "Intracellular Fluid"[Mesh] OR intracellul*[Title/Abstract] OR intra cellul*[Title/Abstract]) AND ("Macrophages"[Mesh] OR macrophag*[Title/Abstract] OR "Monocytes"[Mesh] OR monocyt*[Title/Abstract] OR "Neutrophils"[Mesh] OR neutrophil*[Title/Abstract] OR "Dendritic Cells"[Mesh] OR dendritic cell*[Title/Abstract] OR DC[Title/Abstract] OR "Mast Cells"[Mesh] OR mast cell*[Title/Abstract] OR "Granulocytes"[Mesh] OR granulocyt*[Title/Abstract] OR "Leukocytes"[Mesh] OR leukocy*[Title/Abstract] OR leucocy*[Title/Abstract] OR polymorphonucl*[Title/Abstract] OR "Phagocytes"[Mesh] OR Phagocyt*[Title/Abstract])

1.2. Embase Search String

('antibiotic agent'/exp OR 'anti bacterial agent*':ti,ab OR 'antibacterial agent*':ti,ab OR antibiotic*:ti,ab OR 'bactericidal agent*':ti,ab OR 'anti-biotic*':ti,ab OR 'Anti-microbial drug*':ti,ab OR 'antimicrobial drug*':ti,ab OR 'anti-microbial agent*':ti,ab OR 'antimicrobial agent*':ti,ab OR 'anti-microbial therapy':ti,ab OR 'antimicrobial therapy':ti,ab OR 'Ceftriaxone'/exp OR Ceftriaxon*:ti,ab OR Rocephin*:ti,ab OR 'amoxicillin plus clavulanic acid'/exp OR 'amoxicillin clavulanic acid':ti,ab OR 'amoxicillin/clavulanic acid':ti,ab OR 'Amoxicillin Clavulanate':ti,ab OR Augmentin:ti,ab OR 'Ciprofloxacin'/exp OR Cipro*:ti,ab OR 'Clindamycin'/exp OR Clindamycin*:ti,ab OR 'Dalacin C':ti,ab OR 'gentamicin'/exp OR Gentamicin*:ti,ab OR 'Vancomycin'/exp OR Vancomycin*:ti,ab OR Vancocin*:ti,ab OR 'Penicillin G'/exp OR 'Penicillin G':ti,ab OR Benzylpenicillin*:ti,ab OR 'flucloxacillin'/exp OR Floxacillin*:ti,ab OR Flucloxacillin*:ti,ab OR Floxapen:ti,ab OR 'rifampicin'/exp OR Rifampi*:ti,ab OR Rifadin*:ti,ab OR 'cefazolin'/exp OR Cefazolin*:ti,ab OR Cephazolin*:ti,ab OR Kefzol:ti,ab) AND ('intracellular space'/exp OR 'intracellular fluid'/exp OR intracellul*:ti,ab OR 'intra cellul*':ti,ab) AND ('macrophage'/exp OR macrophag*:ti,ab OR 'monocyte'/exp OR monocyt*:ti,ab OR 'neutrophil'/exp OR neutrophil*:ti,ab OR 'dendritic cell'/exp OR 'dendritic cell*':ti,ab OR DC:ti,ab OR 'mast cell'/exp OR 'mast cell*':ti,ab OR 'granulocyte'/exp OR granulocyt*:ti,ab OR 'leukocyte'/exp OR leukocy*:ti,ab OR leucocy*:ti,ab OR polymorphonucl*:ti,ab OR 'phagocyte'/exp OR Phagocyt*:ti,ab) AND [embase]/lim NOT [medline]/lim

Only “article” and “article in press” (NOT ('chapter'/it OR 'conference abstract'/it OR 'conference paper'/it OR 'conference review'/it OR 'editorial'/it OR 'letter'/it OR 'note'/it OR 'review'/it OR 'short survey'/it)
